# Anatomical precision in the omics era: a proposed framework for selective para-aortic lymphadenectomy in epithelial ovarian cancer

**DOI:** 10.3389/fmed.2026.1883825

**Published:** 2026-07-08

**Authors:** Roli Purwar, Dency Hansalia, Meenu Singh, Anvitha Manoj, Manoj Pandey

**Affiliations:** 1 Department of Surgical Oncology, Institute of Medical Sciences, Banaras Hindu University, Varanasi, India; 2 HTAIn Resource Centre, Institute of Medical Sciences, Banaras Hindu University, Varanasi, India; 3 DHR-ICMR Advanced Molecular Oncology Diagnostic Services (DIAMOnDS), Institute of Medical Sciences, Banaras Hindu University, Varanasi, India

**Keywords:** epithelial ovarian cancer, para-aortic lymphadenectomy, platinum resistance, homologous recombination deficiency, multi-omics, precision surgery

## Abstract

**Objectives:**

The LION and CARACO trials demonstrated that routine systematic pelvic and para-aortic lymphadenectomy (LAD) confers no survival benefit in clinically node-negative advanced epithelial ovarian cancer (EOC), while significantly increasing perioperative morbidity. However, these trials evaluated unselected populations without molecular stratification. This review proposes an omics-guided, anatomically precise surgical paradigm to identify patients who may benefit from selective retroperitoneal clearance.

**Methods:**

A synthesis of recent landmark clinical trials, international surgical guidelines, and emerging multi-omics datasets was conducted to evaluate retroperitoneal lymph nodes as complex architectural niches characterized by passive drug diffusion limits and chemotherapy-induced structural remodelling.

**Results:**

Approximately 30–50% of advanced high-grade serous EOC cases exhibit primary or acquired resistance to standard platinum/taxane chemotherapy. Biological and spatial transcriptomic data indicate that retroperitoneal nodes can act as sanctuaries where resistant clones persist despite systemic therapy, driving later recurrence. Validated preoperative biomarkers; including homologous recombination proficiency (HRD-negative status), investigational markers such as enhancer-associated MSH6 downregulation and specific proteomic panels under investigation (e.g., PRKAR2B/SKP2) show strong predictive potential for primary chemoresistance within exploratory validation cohorts.

**Conclusion:**

A one-size-fits-all approach to surgical de-escalation tends to overlook a critical, molecularly defined minority. Shifting the surgical paradigm toward biomarker-guided anatomical precision offers a conceptual, hypothesis-generating blueprint for future clinical trials, testing whether restricting systematic para-aortic lymphadenectomy to preoperatively identified chemoresistant subsets provides true therapeutic utility.

## Graphical abstract

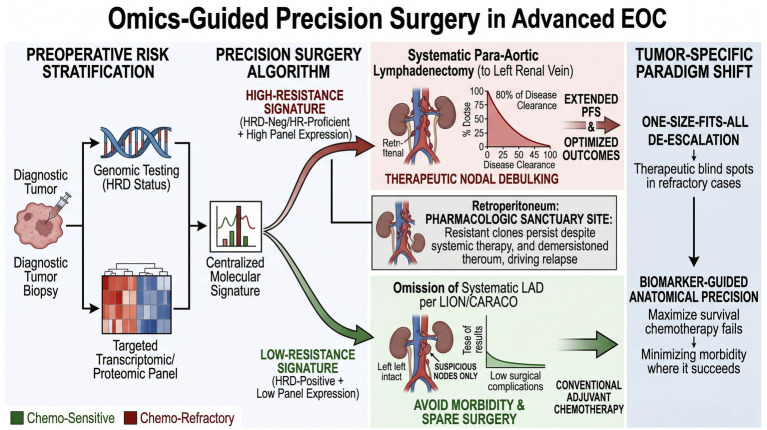

## 1 Introduction

Epithelial ovarian cancer remains the most lethal gynecologic malignancy, with 5-year survival rates below 50% in advanced stages despite optimal cytoreductive surgery and platinum-based chemotherapy (1). Lymphatic spread, particularly to para-aortic nodes, represents a key route of dissemination and occurs in 50–75% of patients with FIGO stage III–IV disease (2–4). Historically, systematic pelvic and para-aortic lymphadenectomy was considered the standard treatment for detecting occult metastasis, accurately staging tumors and guiding decisions regarding adjuvant therapy while also providing potential therapeutic benefit (5, 6). The therapeutic rationale rested on the assumption that removal of occult nodal metastases could reduce tumor burden and prevent relapse (7). Retrospective data and early meta-analyses supported this view, showing improved overall survival (OS) with lymphadenectomy in advanced EOC (3, 8). However, two pivotal randomized controlled trials have fundamentally challenged this practice. The LION trial (9) randomized 650 women with advanced EOC and clinically negative nodes after complete intra-abdominal resection to systematic lymphadenectomy versus no lymphadenectomy. No difference in PFS (median 25.5 vs. 25.5 months) or OS (median 65.5 vs. 69.2 months) was observed, yet serious complications (lymphocysts, infections, re-laparotomy) and 60-day mortality were significantly higher in the lymphadenectomy arm. The CARACO trial (10) extended these findings to the neoadjuvant chemotherapy (NACT) setting, randomizing patients undergoing interval cytoreductive surgery. Again, omission of retroperitoneal lymphadenectomy yielded equivalent PFS and OS with reduced morbidity. Yet a critical limitation unites both trials: they enrolled populations without molecular stratification. EOC is biologically heterogeneous; approximately 30–50% of high-grade serous cases exhibit primary or acquired resistance to platinum/taxane chemotherapy, driven by HRD-negative status, metabolic reprogramming, or specific transcriptomic/proteomic signatures (11–13). In chemo-sensitive tumors, adjuvant therapy effectively controls microscopic nodal disease; in resistant tumors, lymph nodes may function as pharmacologic sanctuaries (14). The omics revolution now offers preoperative tools to identify these high-risk patients, the framework proposed herein is strictly hypothesis-generating; it is designed to inspire future biomarker-stratified clinical trial designs and does not challenge or invalidate the current unselected standard-of-care guidelines established by the LION and CARACO protocols. This review synthesizes the evidence supporting selective para-aortic lymphadenectomy in molecularly defined chemo resistant subsets.

### 1.1 Methodology and literature search strategy

To ensure a transparent and reproducible evaluation of the literature, a comprehensive search was conducted across the PubMed, Embase, and Scopus databases for peer-reviewed articles published up to May 2026. The search strategy utilized combinations of Medical Subject Headings (MeSH) and free-text keywords including: (*“Epithelial Ovarian Cancer”* OR *“Ovarian Neoplasms”*) AND (*“Lymphadenectomy”* OR *“Lymph Node Excision”*) AND (*“Multi-omics”* OR *“Platinum Resistance”* OR *“Homologous Recombination Deficiency”* OR *“Lymphangiogenesis”*).

Inclusion criteria were restricted to prospective randomized controlled trials, retrospective validation cohorts evaluating genomic/proteomic datasets, spatial transcriptomic analyses, and consensus guidelines from international societies (ESGO/ESMO/NCCN). Conceptual or speculative commentaries lacking mechanistic or empirical validation data were excluded. Data synthesis followed a narrative approach designed to contrast Level 1 clinical de-escalation outcomes with translational microenvironmental biology.

## 2 Current evidence and limitations of routine lymphadenectomy

LION and CARACO provide Level 1 evidence against routine systematic lymphadenectomy. In LION, lymph-node positivity was detected in 55.7% of the lymphadenectomy arm despite normal preoperative imaging and intraoperative assessment, yet this did not translate into survival gain (9, 10). CARACO confirmed similar findings post-NACT, with nodal involvement in nearly 50% of cases but no oncologic benefit from resection (10). Meta-analyses incorporating these trials show mixed results. While some observational data suggested OS benefit (RR 1.31 for OS in Ronsini et al. 2024 (8)), pooled randomized evidence demonstrates neutral impact on survival once LION and CARACO are included (4, 15). Morbidity, however, is consistently higher: increased lymphocysts (7–25%), infections, transfusions, and re-interventions (9, 10). ESGO/ESMO/ESP 2025 explicitly advises against systematic lymphadenectomy in advanced disease with non-suspicious nodes, reserving resection for bulky or suspicious nodes only (16). NCCN (17) guidelines also recommend only removal of clinically positive nodes rather than systematic lymphadenectomy in advanced stages because it does not improve survival, rather it is increasing morbidity. Some, retrospective subgroup analyses hint at differential clinical benefit of lymphadenectomy in clear-cell histology or cases with macroscopic nodal disease (3, 18). Unbiased clinical execution demands a clear distinction between biological plausibility and proven therapeutic survival benefit. The documented persistence of macroscopic or microscopic nodal disease following systemic therapy does not automatically dictate that systematic surgical clearance will extend patient survival. If the underlying tumor biology is governed by systemic chemoresistance pathways, localized retroperitoneal cytoreduction may function as a redundant surgical extension. Removing a localized reservoir fails to alter the patient’s ultimate oncologic trajectory if matching chemoresistant clones remain active within the peritoneal cavity or visceral sub-niches. Nevertheless, these recommendations are applied to unselected patients in whom molecular profiling is not done. Consequently, absence of molecular stratification in LION and CARACO leaves open the possibility that the “average” result may mask benefit in chemo resistant minorities.

## 3 The dual-mechanism nodal niche: passive diffusion limitations and induced remodeling

**Figure 1:**
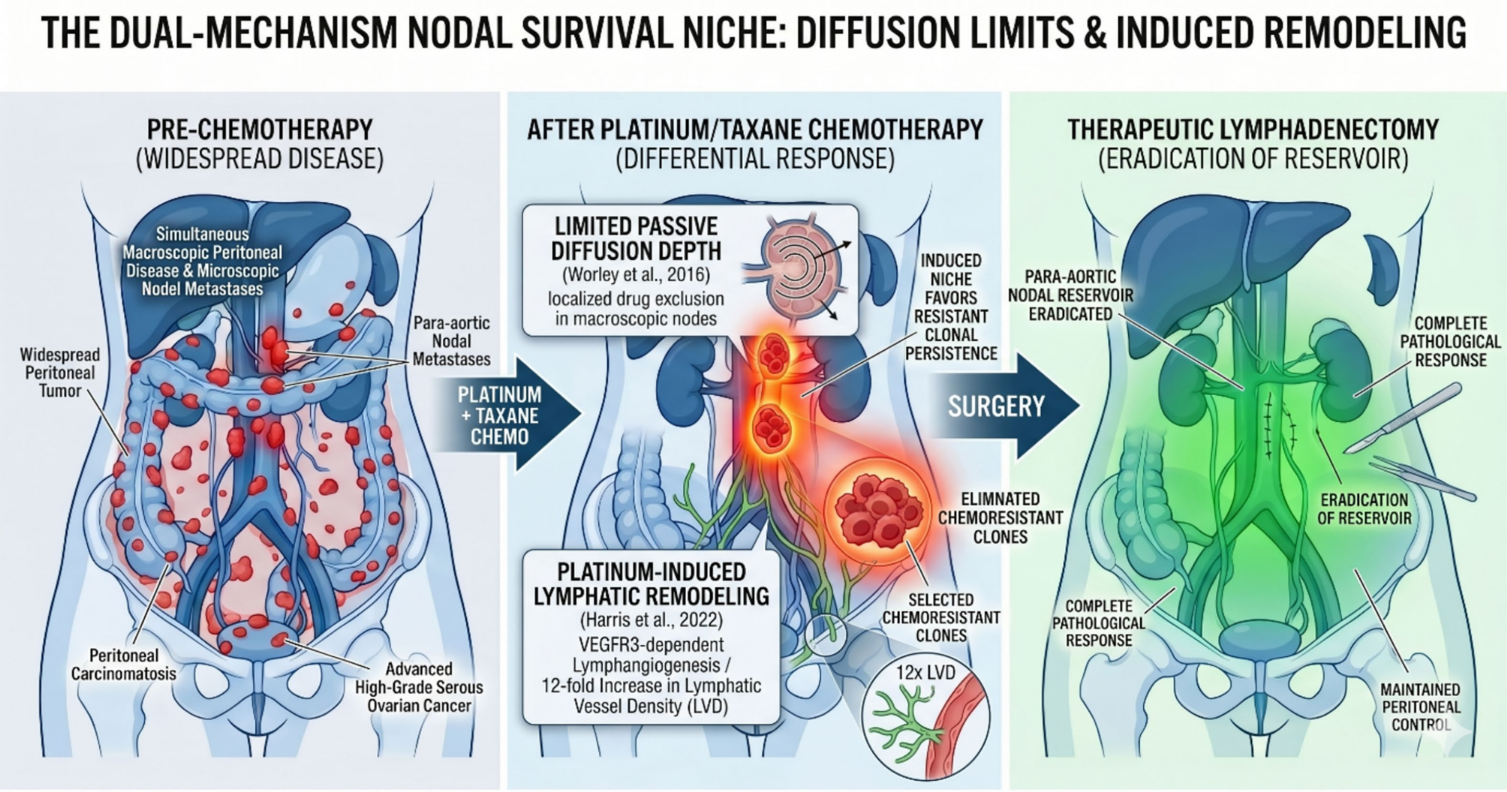
The dual-mechanism retroperitoneal nodal survival niche. Schematic overview of the proposed exploratory framework for a dual-mechanism retroperitoneal nodal survival niche: (Left) Pre-chemotherapy widespread disease. (Middle) Post-chemotherapy nodal persistence mediated by limited passive drug diffusion depth (27) paired with active, platinum-induced VEGFR3-dependent lymphangiogenesis (22) that shields selected chemoresistant clones. (Right) Targeted therapeutic lymphadenectomy to surgically eradicate this remodeled reservoir.

The observed survival of viable tumor tissue within pelvic and para-aortic lymph nodes following neoadjuvant chemotherapy (NACT) has historically been framed as a simple “pharmacologic sanctuary” (19, 20). However, contemporary pharmacokinetic and microenvironmental data reveal a far more complex, dual-layered survival mechanism that balances passive structural diffusion limits with active, therapy-induced tissue expansion (Figure 1).

### 3.1 Pharmacokinetic limitations and diffusion depth

Comparative tracking demonstrates that following standard intravenous (IV) administration, platinum agents like carboplatin equilibrate effectively between the blood plasma and free-flowing lymphatic fluid, achieving comparable total drug exposure (AUC_0-24h_) within the fluid phase (21). However, a critical physiological barrier emerges when evaluating solid tissue penetration depth. Within dense, macrometastatic nodal aggregates, disorganized micro-vascularization and highly elevated interstitial fluid pressure (IFP) severely restrict passive drug transport. Because platinum compounds rely heavily on passive diffusion away from functional capillaries; effectively penetrating only a few cell layers deep, the core environments of large, solid retroperitoneal metastases experience localized drug exclusion and sub-therapeutic platinum levels, allowing deep-seated tumor clones to escape cytotoxic clearance.

### 3.2 Platinum-induced structural remodeling

This passive anatomical shielding is actively augmented by systemic chemotherapy itself. Recent translational evidence demonstrates that platinum exposure does not leave the lymphatic endothelium inert; rather, carboplatin directly induces a robust, VEGFR3-dependent lymphangiogenic cascade and compromises lymphatic endothelial cell (LEC) junctions (22). In patients with advanced high-grade serous ovarian cancer (HGSOC) undergoing neoadjuvant platinum regimens, treated tissues demonstrate a striking 12-fold increase in lymphatic vessel density (LVD) compared to untreated controls (22).

This dual mechanism of passive diffusion limitation and active structural remodeling transforms the local lymphatic network into a highly protected pharmacologic sanctuary. From a translational perspective, this microenvironmentally altered niche cannot be effectively addressed by treating lymph nodes as isolated, stochastic downstream filters spread across broad, arbitrary geometric boundaries—the historical rationale behind unselected systematic lymphadenectomy.

Instead, these biological sanctuary dynamics reinforce the modern surgical paradigm of the ‘regional cancer field’ (23). Because the primary tumor and its first- and second-line lymphatic basins develop from a shared, embryologically distinct topogenetic compartment, the induced remodeling and sub-clonal escape are structurally confined within this specific ovarian lymphatic basin (24). Conceptualizing the nodal niche as a defined regional cancer field rather than an open systemic pathway provides the necessary anatomical rationale to match our molecular stratification model; it justifies why surgical clearance must be highly focused and radical within the tumor’s developmental field, while safely sparing unaffected compartments.

This active expansion and structural rewiring of the lymphatic network, combined with the tissue diffusion limits of the macrometastatic core, along with distinct metabolic and immune invasion profile and regional cancer field may transform the retroperitoneal node into a highly dynamic, therapy-protected survival niche (25, 26) (Figure 1).

## 4 Omics-driven identification of platinum/taxane resistance

**Figure 2:**
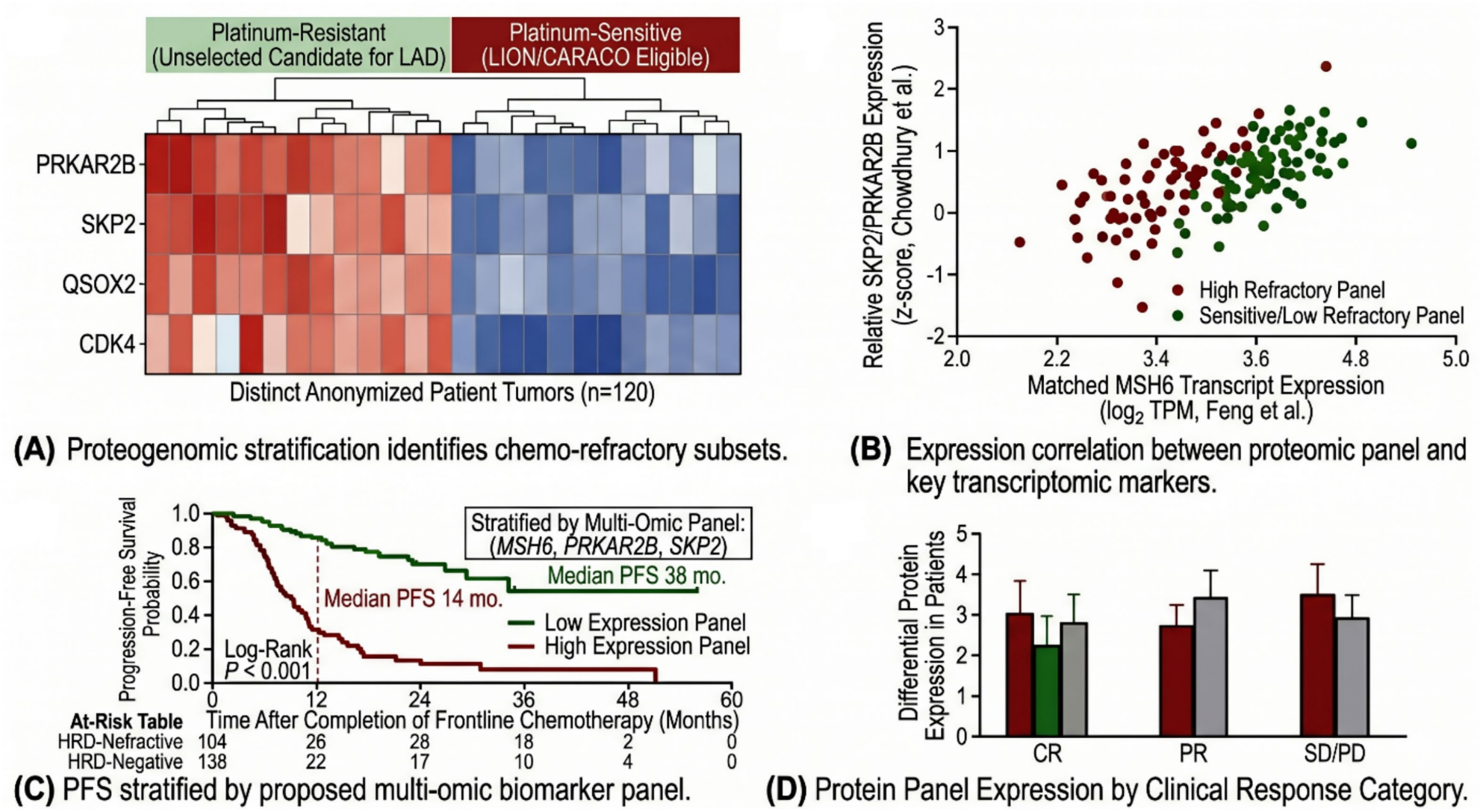
Multi-omics stratification model (Hypothesis-generating data template). **(A)** Proteogenomic clustering heatmap displaying standardized relative protein expression patterns (z-scores) of PRKAR2B, SKP2, QSOX2, and CDK4, effectively stratifying distinct patient tumors into platinum-resistant and platinum-sensitive cohorts. **(B)** Cross-omics scatter plot demonstrating the significant expression correlation between the targeted proteomic panel (SKP2/PRKAR2B) and matched MSH6 transcript levels across patient samples. **(C)** Kaplan–Meier curves displaying a stark divergence in progression-free survival (PFS) between the high-expression and low-expression multi-omic biomarker cohorts (*p* < 0.001). **(D)** Categorical expression analysis showcasing the progressive up-regulation of the target protein panel across worsening clinical response categories (Complete Response vs. Stable/Progressive Disease).

The omics era has transformed our ability to predict chemoresistance preoperatively. HRD status is the most clinically mature biomarker. HRD-positive tumors (BRCA1/2 mutated or genomic instability score ≥42) demonstrate superior platinum sensitivity and PARP-inhibitor benefit (28, 29) (Figure 2). Conversely, HR-proficient (HRD-negative) tumors, comprising 30–40% of high-grade serous EOC, are classically platinum-resistant and derive less benefit from standard regimens (30, 31). Beyond clinically validated HRD testing, exploratory multi-omics signatures offer higher resolution in preclinical models. For instance, Song et al. (12) identified enhancer-associated MSH6 downregulation as a potential multi-omics predictor. Similarly, highly experimental proteomic panels under investigation (e.g., PRKAR2B, SKP2) have been shown to achieve AUC 0.85–0.96 for resistance prediction within retrospective validation cohorts (12), though they strictly lack prospective clinical validation. Proteomic panels under investigation (e.g., PRKAR2B, SKP2, PSME4, QSOX2, HS2ST1, EFL1) have been shown to achieve AUC 0.85–0.96 for resistance prediction in validation cohorts (32). Transcriptomic markers (AKIP1, MARVELD1, AKIRIN2) and metabolic/immune signatures further stratify risk (25, 33) (Table 1). These biomarkers are obtainable from diagnostic biopsy or circulating tumor DNA, enabling preoperative decision-making.

**Table 1:** Evaluated omics biomarkers for preoperative identification of platinum/taxane resistance in EOC.

Omics layer	Target biomarkers and molecular signatures	Predictive performance metrics	Direct clinical implication	Key source reference
Genomics	HRD-negative/HR-proficient status; *CCNE1* gene amplification	HR 0.5–0.6 for PFS/OS post-chemotherapy	Primary platinum resistance; strong candidate for systematic LAD, not validated	Feng 2023 (28), Takaya 2020 (29)
Transcriptomics	*MSH6* expression dysregulation; *AKIP1*/*MARVELD1* upregulation	Validation Cohort AUC: 0.70–0.80	Accelerated mismatch repair and apoptotic evasion, not validated in clinical trials	Song 2026 (12), Fekete 2020 (33)
Proteomics	*Increased PRKAR2B*, *SKP2*, *QSOX2* (6-protein signature panel)	External Validation AUC: 0.855–0.964	Preoperative identification of chemo-refractory tumors, not validated in clinical trials	Chowdhury 2023 (32)
Multi-omics	Macropinocytosis, lipid catabolism, and JAK1/STAT pathway activation signatures	Strong correlation with decreased Platinum-Free Interval (PFI)	Identifies metabolic and stromal sanctuary-site biology, need clinical validation	Zheng 2026 (25), Suzuki 2025 (45)

It is essential to contextualize these surgical hypotheses within contemporary, first-line systemic maintenance algorithms. While standard cytotoxic regimens show lower efficacy in Homologous recombination proficient (HRD-negative) subset, standard-of-care maintenance strategies, such as single-agent bevacizumab derived from GOG-0218 and PAOLA-1, emerging combinations with immune checkpoint inhibitors, and modern Antibody-Drug Conjugates (ADCs) like Mirvetuximab soravtansine for folate receptor alpha (FRα)-positive tumors—continue to rapidly expand treatment options. Consequently, any exploratory proposal to escalate retroperitoneal surgical radicality in HRD-negative disease cannot be evaluated in isolation; it must be viewed as an investigational surgical adjunct meant to be tested alongside, and integrated into, the contemporary systemic maintenance backbone.

## 5 Proposed molecular mechanism of chemoresistance in EOC

The development of platinum and taxane resistance in epithelial ovarian cancer (EOC) is a highly complex, multi-layered process involving an interplay of genomic, transcriptomic, proteomic, and metabolic adaptations (34). At the genomic level, alterations in DNA repair pathways play a central role. While homologous recombination deficient (HRD) tumors are highly sensitive to DNA cross-linking platinum agents, homologous recombination proficient (HR-proficient) tumors retain intact DNA repair capabilities, allowing them to repair platinum-induced DNA adducts and survive cytotoxic therapy (35). This genomic resistance is frequently compounded by specific gene copy number alterations, notably *CCNE1* amplification, which accelerates the cell cycle transition from G1 to S phase, promoting uncontrolled proliferation and primary chemoresistance in high-grade serous phenotypes (36).

**Figure 3:**
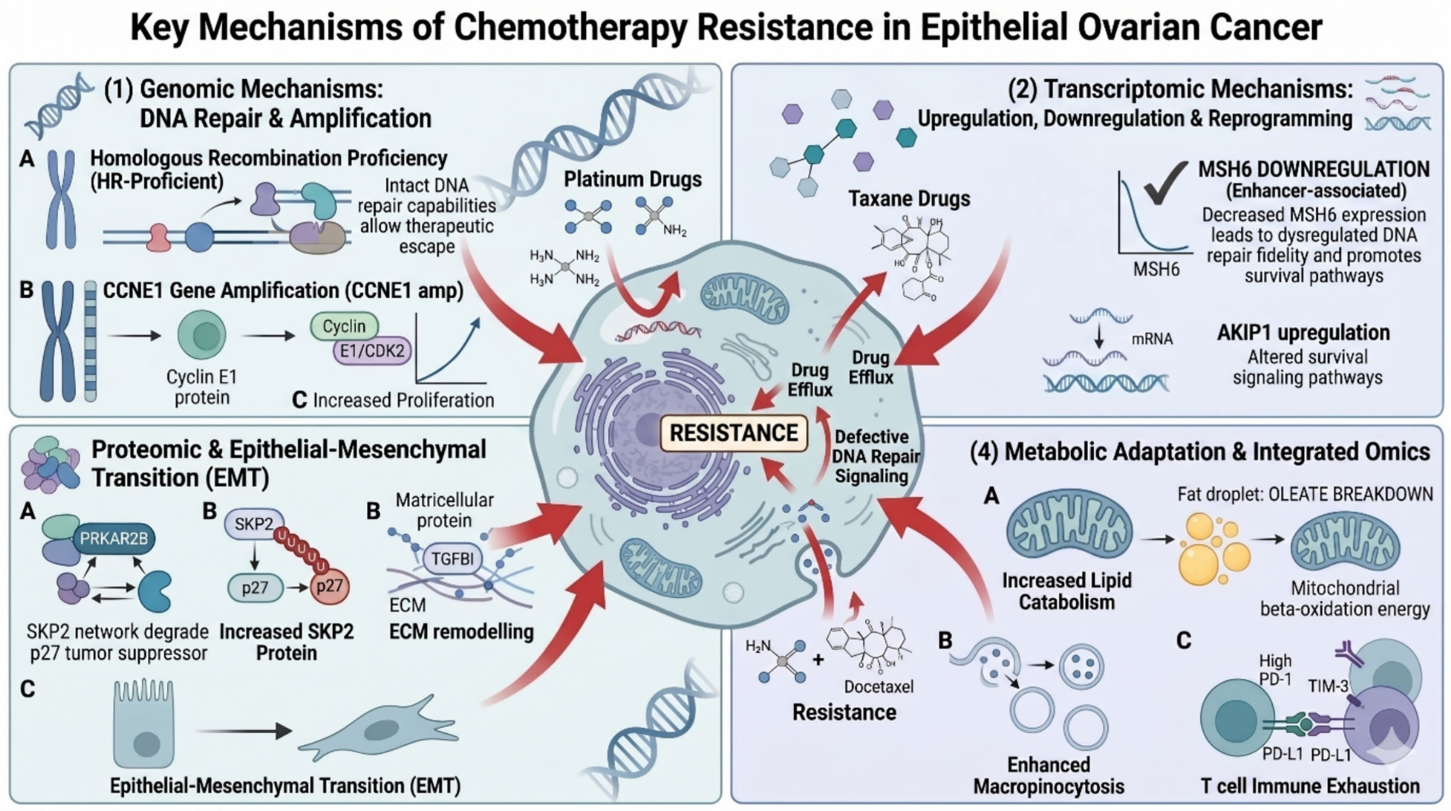
Key molecular and microenvironmental mechanisms of chemotherapy resistance in EOC. Summary of the cross-talk driving therapeutic escape, including: (1) genomic alterations and CCNE1 amplification; (2) transcriptomic reprogramming of mismatch repair; (3) proteomic up-regulations (PRKAR2B/SKP2) inducing epithelial-mesenchymal transition (EMT); and (4) metabolic shifts toward active lipid catabolism alongside T-cell immune exhaustion within the tumor niche.

At the transcriptomic and proteomic levels, tumors reprogram key signaling cascades to minimize drug accumulation and promote cell survival. Alterations in drug influx and efflux pathways significantly reduce intracellular platinum concentrations (34). This is characterized by the downregulation of copper transporter 1 (CTR1); which facilitates cisplatin entry, coupled with the upregulation of ATP-binding cassette transporters, such as multidrug resistance proteins 2 and 4 (MRP2/4), and the exporters ATP7A and ATP7B, which actively sequester and expel platinum salts from the cell (34). Concurrently, specific transcriptomic signatures, such as the upregulation of *AKIP1* or the enhancer-associated dysregulation of mismatch repair genes like *MSH6*, modulate downstream cell-survival pathways and blunt the apoptotic signals typically triggered by DNA damage (36). Proteomic profiling further reveals that overexpressed protein complexes, such as the *PRKAR2B* and *SKP2* network, actively degrade key tumor suppressors (like p27), bypassing checkpoint-mediated apoptosis (Figure 3).

Finally, metabolic adaptation and microenvironmental influences create a highly protective niche for resistant cells. Multi-omics analyses indicate that chemo resistant EOC cells undergo extensive metabolic reprogramming, shifting toward increased lipid catabolism and mitochondrial beta-oxidation to fulfill the high energy demands required for cell survival under chemotherapeutic stress (11). This is frequently accompanied by cellular adaptations like active macropinocytosis, which allows tumor cells to scavenge extracellular nutrients within the retroperitoneal or ascitic microenvironment (11). Within this localized nodal microenvironment, these metabolic shifts directly interact with the tumor stroma to recruit immunosuppressive cells, generating an exhausting immune microenvironment characterized by high expression of immune checkpoints like PD-L1 and TIM-3, which effectively shields the lingering chemoresistant clones from host immune surveillance (11).

### 5.1 Histology-specific deviations in nodal biology

Advanced epithelial ovarian cancer encompasses distinct disease entities with vastly different biological profiles; therefore, generalizing a retroperitoneal surgical model across all histologies presents significant clinical risks. The landmark LION and CARACO trials enrolled populations overwhelmingly dominated by High-Grade Serous Ovarian Carcinoma (HGSOC). Extrapolating these concepts to non-serous histologies requires extreme caution:

- Clear Cell Carcinoma (CCC): Classically displays an intrinsic HRD-negative/HR-proficient status and marked primary resistance to platinum agents, yet its lymphatic dissemination pathways and metabolic drivers differ fundamentally from HGSOC.
- Low-Grade Serous Carcinoma (LGSOC): Driven primarily by indolent MAPK pathway alterations (e.g., KRAS/BRAF mutations). These tumors are inherently insensitive to standard cytotoxic chemotherapy; thus, postoperative nodal persistence reflects baseline chemo-insensitivity rather than active microenvironmental selection.
- Mucinous and Endometrioid Carcinomas: Exhibit distinct metastatic cascades and significantly lower baseline rates of occult retroperitoneal involvement.

Therefore, the proposed omics-guided framework must be strictly constrained to HGSOC models until dedicated histology-specific validation cohorts are established.

## 6 Proposed precision-surgery algorithm

**Figure 4:**
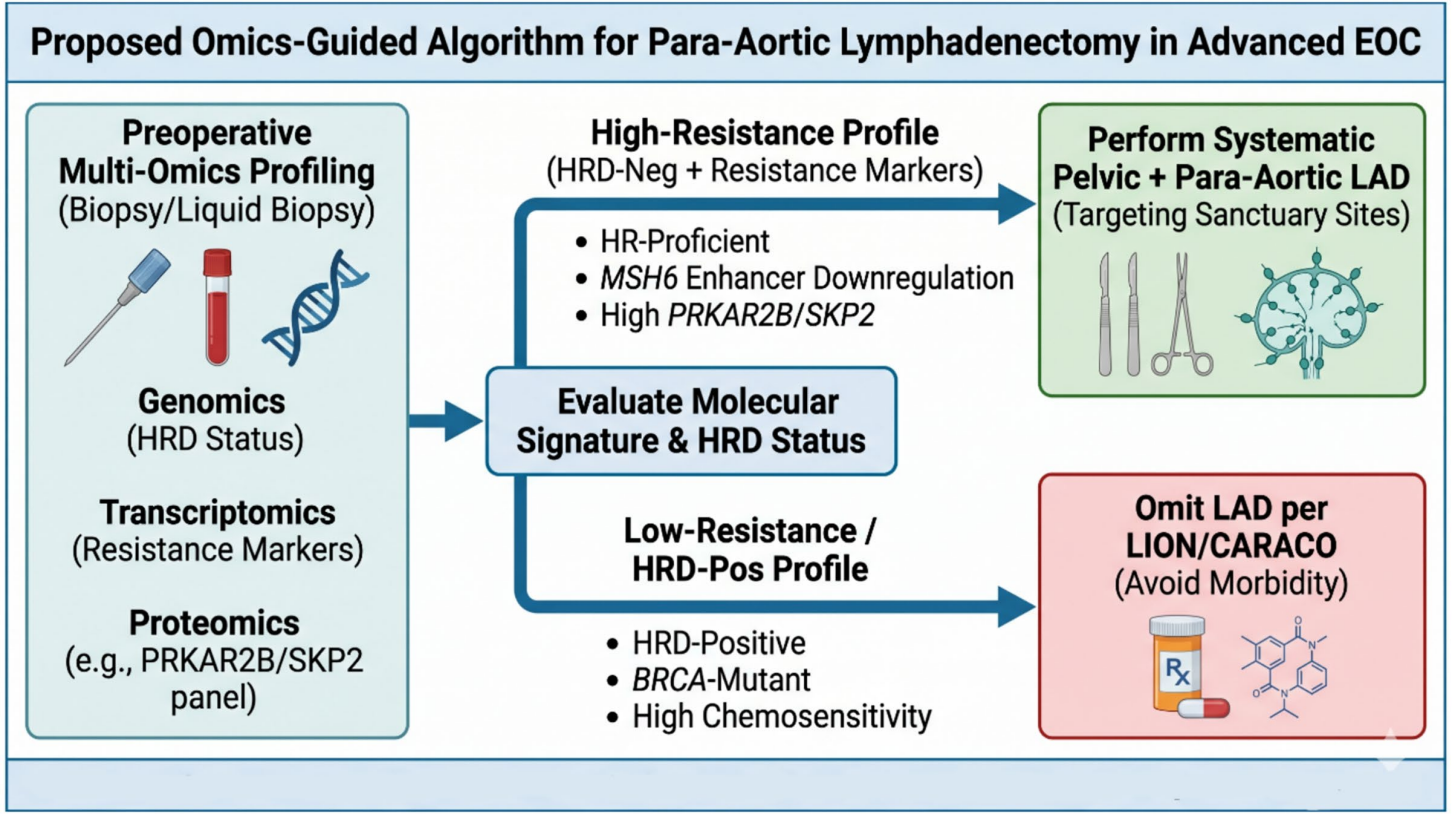
Proposed exploratory omics-guided surgical algorithm for para-aortic lymphadenectomy. This decision workflow represents a conceptual blueprint for prospective clinical trial stratification and is not intended for current clinical practice.

To operationalize these biological insights into clinical practice, we propose a structured, biomarker-guided surgical workflow for patients with advanced epithelial ovarian cancer undergoing planned primary or interval complete cytoreduction. Rather than applying a uniform approach to retroperitoneal management, patients are stratified preoperatively using a comprehensive multi-omics profiling panel obtained via diagnostic tissue biopsy or circulating tumor DNA (liquid biopsy). This assessment integrates genomic homologous recombination deficiency (HRD) testing with a targeted transcriptomic and proteomic resistance marker panel to effectively map the tumor’s therapeutic vulnerability (Figure 4).

Under this algorithm, patients demonstrating a high-resistance signature; defined as an HR-proficient (HRD-negative) status combined with the upregulation of two or more validated resistance markers like the enhancer-associated downregulation of MSH6, which reduces DNA mismatch repair fidelity and allows tumor clones to evade damage-induced apoptosis, could theoretically be triaged toward investigational retroperitoneal clearance within a clinical trial framework. In this specific cohort, where standard systemic therapies are highly likely to fail, a systematic pelvic and para-aortic lymphadenectomy a focused, biology-driven regional cancer field surgery targeting the defined first- and second-line nodes of the ovarian basin is proposed as a therapeutic strategy to be evaluated.

Conversely, patients presenting with a low-resistance or HRD-positive/BRCA-mutated profile are directed toward surgical de-escalation. Because microscopic nodal disease in this subset remains highly chemo sensitive and is effectively controlled by conventional adjuvant systemic therapies and maintenance therapies, systematic retroperitoneal dissection can be safely omitted in accordance with the level-1 evidence established by the LION and CARACO trials (9, 10). For these highly sensitive patients, surgical intervention in the retroperitoneum is strictly limited to the resection of macroscopically suspicious or bulky nodes, thereby sparing them from the extensive perioperative morbidity associated with radical lymphadenectomy.

This preoperative framework can be dynamically refined in the operating room. The integration of intraoperative frozen sections or rapid molecular feedback loops offers a real-time mechanism to clarify ambiguous cases or confirm nodal pathology on the table. Furthermore, when systematic lymphadenectomy is indicated for the high-resistance cohort, utilizing minimally invasive surgical platforms or utilizing an extraperitoneal approach can be selectively deployed to substantially mitigate surgical morbidity, minimize recovery times, and avoid delaying subsequent lines of systemic therapy.

While direct evidence from biomarker-stratified randomized trials in ovarian cancer is currently lacking but the proposed argument for selective para-aortic lymphadenectomy in molecularly defined chemo resistant EOC is supported by convergent indirect data: (i) absence of benefit from routine LAD in unstratified populations (LION (9), CARACO (10)), (ii) robust multi-omics predictors of platinum/taxane resistance that are already clinically available but yet to be validated (33, 37–39), and (iii) persistent nodal disease as a recognized sanctuary for resistant clones (19, 20, 40). This paradigm mirrors the successful shift toward precision medicine in other solid tumors also and provides a clear rationale for the next generation of surgical trials (41, 42).

## 7 Potential benefits, risks, and counter-arguments

*Benefits*: In the resistant subset, nodal clearance could reduce sanctuary-site relapse, improve PFS/OS (supported by protective meta-analytic signals in unselected cohorts) (8); and allow earlier escalation to clinical trials of non-platinum agents or antibody-drug conjugates. Accurate nodal staging also informs prognosis and adjuvant strategy.

*Risk mitigation*: Limiting lymphadenectomy to the chemo resistant fraction minimizes population-level morbidity compared with routine practice (43). Modern techniques (extraperitoneal access, energy devices) further reduce complications. Cost of omics testing is offset by avoiding relapsed and resistant cases, re-surgeries and multiple lines of chemotherapies and targeted agents with improved patient outcomes. *Counter-arguments*: No direct Level 1 data exist for omics-stratified lymphadenectomy, a limitation acknowledged here. However, convergent indirect evidence provides a compelling hypothesis, that needs to be tested. This mirrors successful precision shifts in breast and colorectal cancer.

## 8 Future directions

**Figure 5:**
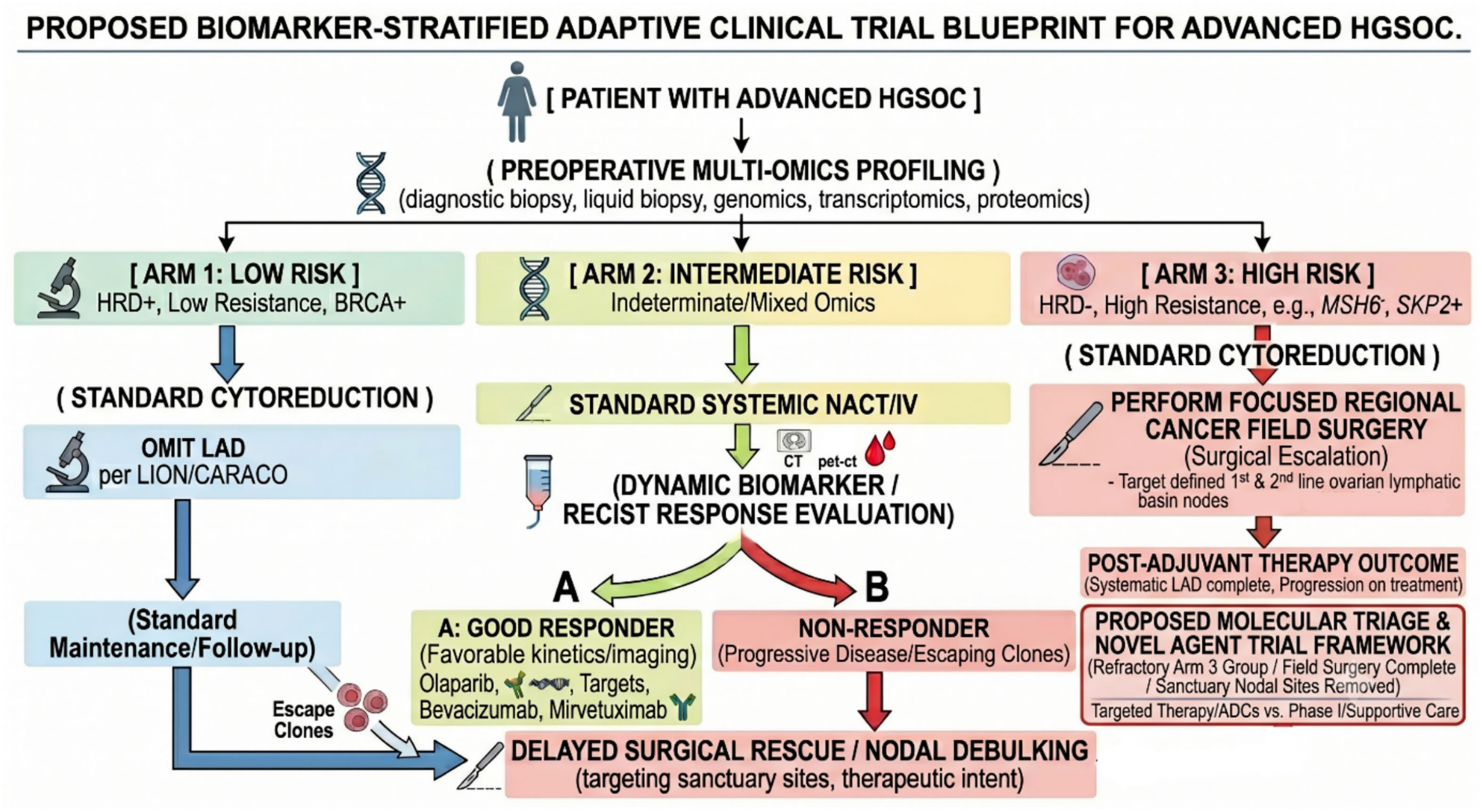
Proposed adaptive, biomarker-stratified clinical trial blueprint for advanced HGSOC. Following preoperative multi-omics profiling, patients are stratified into Low (Arm 1: de-escalation), Intermediate (Arm 2: adaptive response triage), and High (Arm 3: surgical escalation) risk cohorts based on HRD status and resistance markers. Arm 3 receives upfront systematic lymphadenectomy with subsequent triage of refractory cases to novel agent trials via longitudinal molecular re-profiling rather than delayed surgery.

To successfully transition from the current unselected, “one-size-fits-all” surgical approach to proposed biomarker-driven anatomical precision, the next generation of clinical trial designs must be fundamentally restructured. Moving forward, prospective randomized controlled trials must prioritize front-line molecular stratification, specifically isolating high-risk cohorts such as homologous recombination proficient (HRD-negative) advanced epithelial ovarian cancer (EOC) to definitively measure the survival impact of systematic retroperitoneal debulking (Figure 5).

Beyond standard tissue genomics, the integration of advanced diagnostic modalities offers a multi-dimensional framework to enhance preoperative accuracy. Incorporating high-throughput radiomics and artificial intelligence-driven pathomics can uncover macroscopic and microscopic spatial features of tumor heterogeneity and nodal involvement that escape conventional imaging (43, 44). Concurrently, validating real-time liquid biopsies and tracking circulating tumor DNA kinetics can provide non-invasive longitudinal profiling of clonal evolution, catching the emergence of treatment-resistant sub-clones before interval cytoreduction. Furthermore, while sentinel lymph node mapping has demonstrated high sensitivity and low morbidity in early-stage disease, adapting these targeted lymphatic mapping concepts into advanced-stage cytoreductive protocols warrants rigorous evaluation to determine if a more localized, biopsy-guided approach can safely replace full retroperitoneal clearance in boundary-line cases.

In conclusion, the omics revolution challenges the surgical oncology community to abandon legacy, non-selective de-escalation pathways in favor of biological customization. Restricting systematic para-aortic lymphadenectomy exclusively to molecularly defined chemo resistant cohorts may offer a balanced, rational approach to advanced EOC management. This paradigm shifts the operative objective from traditional staging to targeted, therapeutic surgery, maximizing survival potential where standard systemic options fail while effectively sparing chemo sensitive patients from unnecessary, morbid surgical extensions. Aligning surgical radicality with the distinct biological profiles of individual tumors represents the most promising frontier in improving outcomes for this historically recalcitrant patient subset and needs to be tested.

## 9 Conclusion

The management of the retroperitoneum in advanced epithelial ovarian cancer has reached a critical evolutionary crossroads. While landmark clinical trials have successfully driven a necessary de-escalation of routine, non-selective surgery to safeguard patient quality of life, a standardized “one-size-fits-all” abandonment of lymphadenectomy may create a therapeutic blind spot. It inadvertently may overlook possibility of a distinct biological subpopulation whose disease biology may utilize the retroperitoneal lymph nodes as a pharmacologic sanctuary site against standard cytotoxic regimens.

The precision oncology requires a symmetrical paradigm shift where surgical execution is as molecularly tailored as systemic therapy. Proposed replacement of rigid anatomical dogmas with preoperative multi-omics profiling, the surgical oncologist can transition from empirical resections to biomarker-guided interventions. Restricting systematic pelvic and para-aortic lymphadenectomy exclusively to preoperatively identified, chemo resistant tumors redefine the operation as a targeted, high-impact therapeutic tool. This balanced framework may maximize survival opportunities for patients facing primary chemoresistance while successfully shielding highly chemo sensitive individuals from unnecessary surgical morbidity. Ultimately, integrating molecular stratification into the surgical decision-making process reconciles operative radicality with the biological heterogeneity of ovarian cancer, and has a potential to establish a new standard for modern gynecologic oncology.
